# Distribution of estrogen receptor in the rabbit cervix during pregnancy with special reference to stromal elements: an immunohistochemical study

**DOI:** 10.1038/s41598-020-70323-4

**Published:** 2020-08-12

**Authors:** Fatma El-Zahraa A. Mustafa, Ruwaida Elhanbaly

**Affiliations:** Department of Anatomy and Histology, Faculty of Vet. Medicine, Assiut University, Assiut, 71515 Egypt

**Keywords:** Cell biology, Immunology, Physiology, Structural biology

## Abstract

The estrogen plays a critical role during pregnancy through their receptors. Although the rabbit is one of the most important lab animal estrogen receptor alpha (ERA) localization on basic cells, newly discovered cells including telocyte and neuroendocrine cells, vascular compartments and interstitium during pregnancy not been described. At 0 day pregnancy, the most prominent immunoreactivity was moderate to ERA and observed on the ciliated cells, secretory cells, blood plasma, and interstitium. The smooth muscles and the endothelial cells showed mild immunoreactivity to ERA. Lymphocytes only exhibited strong immunoreactivity to ERA. At 7 days pregnancy moderate immunoreactivity to ERA observed on ciliated cells, secretory cells, smooth muscles, interstitium, and lymphocytes. Strong immunoreactivity to ERA detected on endothelial cells and blood plasma. At 14 days of pregnancy, the most prominent immunoreactivity was strong and detected on ciliated cells, smooth muscles, lymphocytes, and interstitium. Moderate immunoreactivity detected on endothelial cells and blood plasma. Secretory cells only exhibited mild immunoreactivity to ERA. At 21 days of pregnancy, the immunoreactivity to ERA ranged between mild on ciliated cells, smooth muscles, blood plasma and interstitium and negative on secretory cells, endothelial cells and lymphocytes. Our results indicated that the frequency and intensity of ERA immunostaining in the rabbit cervix varied on different structural compartments of the cervix during different pregnancy stages.

## Introduction

The cervix is that the boundary structure positioned between the uterus and the vagina and plays a key role in the pregnancy maintenance and timing of parturition^1^. In addition, the cervix acts as a barrier that protects the upper reproductive tract of the females^2^. The cervix is a dynamic structure with a high capacity to adapt to different events on the female genital tract and acting as a barrier to retain the fetus during pregnancy and at end of pregnancy dilating to allow a normal delivery. Moreover, the cervix has differential biological responses to modifications to the hormonal milieu^3^.

The main target of steroid hormones is the female genitalia. Control of steroid receptors in the cervix is identical to that in the different portions in the genital tract including stimulation and inhibition with estrogen and progesterone respectively^4^.

Moreover, female genitalia development and fertility are regulated by estrogen^5–7^. The cervix is a steroid-dependent organ and is a target for the action of estrogen^8–12^. estrogen receptors normally present on the cervix^13^. Abnormalities detected in the genital tract of knock-out mice for estrogen receptor despite of its normal appearance^14^.

The physiological action of estrogen is obtained via intracellular estrogen receptors^15^. Consequently, hormone action depends on not only the hormone amount but also the number of receptors is very important. So, studying of estrogen receptors distribution at various cervical parts was very important to understand changes that occurred on the various constituent of the cervix during the pregnancy and why cells react on the different manner with similar stimuli of hormone^16^. Two types of ER have been observed in mammals: estrogen receptor alpha (ERA) and estrogen receptor beta (ERβ). Moreover, ERA plays a major role in the different important physiological events of the female genitalia^17,18^. ERA considered the most common type of ER responsible for estrogen action on the cervix^19,20^. During pregnancy cervical remodeling mediated through estrogen^21^.

Different kinds of important cells described in different parts of the cervix and performed various functions. The lining epithelium of the cervix consisted mainly of ciliated cells and secretory cells which play an important role in the initiation and maintenance of pregnancy through cervical mucous secretion. Ciliated cells characterized by numerous long cilia at the apical cell border and secretory cells showed secretory product at apical part of the cells and forming blebs^22^. Also, lymphocytes described at the cervix as part of immune system adaption for pregnancy. Lymphocytes identified as rounded cells with little cytoplasm and large rounded nucleus^22,23^. In addition, telocyte which newly described interstitial cell characterized by small cell body and thin, long cytoplasmic processes. Telocyte plays a precious role in cellular communication and tissue regeneration and observed at different organs of different animals including the genitalia during pregnancy^24–26^. Moreover, neuroendocrine cells which described previously on different organs like the lung and gastrointestinal tract and genital tract. Neuroendocrine cells detected solitary or in clusters and secret hormones. Also, Neuroendocrine cells located toward the luminal border or basal border of the epithelium^27–29^.

There were no available previous studies on the expression of ERA in rabbit cervix during pregnancy despite its importance in meat production and as a laboratory animal. So, we aim to show the expression of ERA in the rabbit cervix at different stages of pregnancy. In addition, we described different cellular constituents as ciliated cells, secretory cells, intraepithelial lymphocyte, neuroendocrine cells, telocyte, smooth muscle fibers, blood cells, endothelial cells, and mesothelial cells. Moreover, ERA expression was demonstrated on interstitium and blood plasma. In addition, this work is a part of the project aimed to study the female genitalia during pregnancy^30^.

## Material and methods

The current research was performed according to the Egyptian laws and guidelines of University for animal care. Faculty of Veterinary Medicine National Ethical Committee, Assiut University, Egypt, has authorized all the steps in the present work.

### Sample collection

The study was approved by the Committee of Use and Care of experimental animals of Faculty of Veterinary Medicine, Assiut University, Egypt. The cervix was collected from the female genitalia of 12 healthy New Zealand white rabbits does (3 for each of the following pregnancy days) at 0, 7, 14 and 21 days of pregnancy. The day of mating is considered the zero day of pregnancy and then we collected the cervix of the pregnant does after 7 days, 14 and 21 days. we detected the pregnant does by palpation method and ultrasonography. The rabbit cervix was dissected immediately after slaughtering and fixed in Bouin’s solution.

### Immunohistochemistry

Paraffin sections of (5 µm) were dewaxed by xylene, rehydrated by ascending grades of alcohol and rinsed by PBS pH 7.4 (3 times for 5 min). Endogenous peroxidase was suppressed by using hydrogen Peroxide block at room temperature. The sections were thoroughly washed by running tap water for an additional 10 min. To enhance antigen retrieval, the slides were treated with 10 mm sodium citrate buffer (pH 6.0) at temperature reached 95–98 in a water bath for 20 min. The sections were cooled for 20 min at room temperature and subsequently were washed in PBS (pH 7.4, 3 times for 5 min). Block non-specific background staining was performed by using Ultra V block, for 5 min at room temperature. Ultra V block application did not exceed 10 min to avoid staining artifact). The primary antibody was applied to the sections overnight at 4 °C. The used primary antibody was a mouse anti-rabbit antibody against Estrogen receptor alpha (ERA) Rabbit (PolyClonal; Cat. #RM-9101-S0 From ThermoFisher Scientific/Lab Vision) at dilution (1:200) in the PBS for one hour at room temperature. Sections were washed using PBS (at pH 7.4, 3 times for 5 min).

The biotinylated secondary antibody was applied for 10 min at room temperature. The Biotinylated secondary antibody was Biotinylated goat Anti-Polyvalent, Anti-mouse igg + Anti -Rabbit igg, Thermo Fisher Scientific, The UK. Lab Vision Corporation, ready to use. Sectioned were washed by PBS (pH 7.4, 3 times for 5 min) and subsequently incubated with streptavidin- peroxidase complex. Thermo Fisher Scientific, UK. LabVision corporation; USA) for10 min at room temperature. Visualization of the bound antibodies was performed using 1 drop of DAB plus chromogen to 2 mL of DAB plus substrate. The mixture was applied and incubated at room temperature for 5 min. The incubation processes were carried out in a humid chamber. Harris hematoxylin was used as counters stained for 30 s. The sections were dehydrated using ethanol and isopropanol I and II, cleared in xylene and covered by DPX^31,32^.

The expression of ERA in the cervix was examined microscopically using OLYMPUS BX51 microscope and the images were taken using OLYMPUS DP72 camera adapted to the microscope. By assessing the intensity of the immunostaining, the staining of the nucleus and/or cytoplasm by the following amount and color of immunostaining: strong (dark brown to black), moderate (brown), mild (light brown) and negative immunostaining (no immunoreactivity)^33^.

Percentage of immunohistochemical positive cells for ERA at different stages of pregnancy were measured using Image–J software. The measurements were carried on 9 section per animal (from each section 3 different areas were measured). Collected data were subjected to the Analysis of Variance (ANOVA) using Statistical Analysis System (SAS). Data were depicted as means ± SEM to show mean data deviation.

### Ethical approval and consent to participate

The study was approved by the Ethics Committee of Assiut University, Egypt.

## Results

Immunohistochemical reactivity described on cervical lining epithelium, intraepithelial lymphocyte, neuroendocrine cells, smooth muscle fibers, blood vessels, lymph vessels, interstitium and different kinds of connective tissue elements including fibroblast, lymphocytes, and telocyctes.

### At 0 day of pregnancy

**Figure 1:**
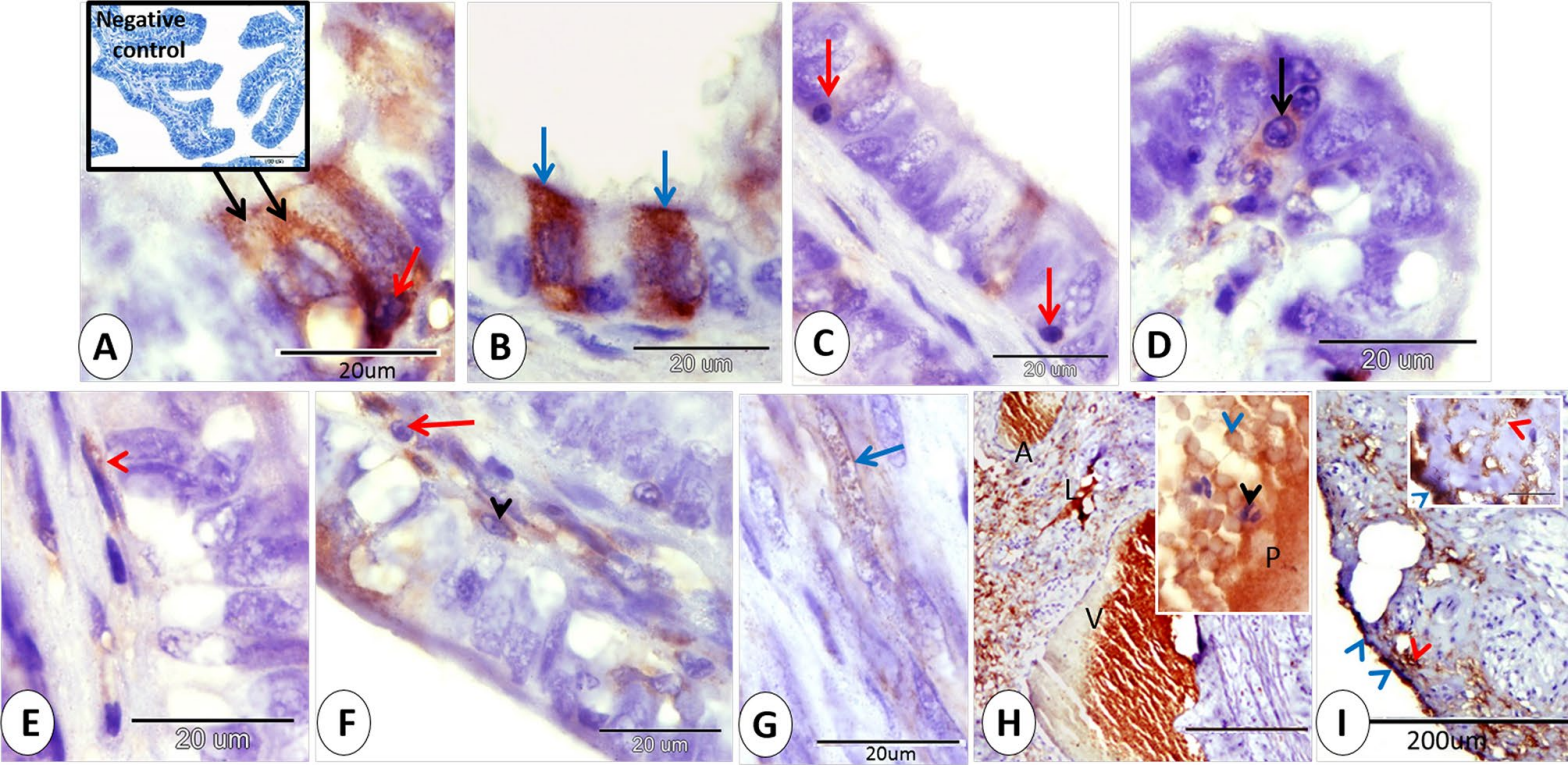
Immunohistochemical localization of ERA in rabbit cervix at 0 day of pregnancy. (**A**) Secretory cells (black arrows) show moderate cytoplasmic and moderate nuclear immunostaining for ERA and Intraepithelial lymphocytes show strong immunostaining for ERA (red arrow). (**B**) Ciliated cells (blue arrows) show moderate cytoplasmic and moderate nuclear immunostaining for ERA. (**C**) Intraepithelial lymphocytes (red arrows) show mild immunostaining for ERA. (**D**) Intraepithelial lymphocytes (black arrows) show moderate immunostaining for ERA. (**E**) Subepithelial telocytes (red arrowhead) show moderate cytoplasmic and negative nuclear immunostaining for ERA. (**F**) fibroblasts (black arrowhead) and lymphocytes (red arrow) showed mild cytoplasmic and negative nuclear ERA immunostaining. (**G**) Smooth muscle fibers (blue arrow) show mild cytoplasmic and mild nuclear immunostaining for ERA. (**H**) the blood plasma (P), red blood cell (blue arrowhead) and cytoplasm of leukocyte (black arrowhead) within the arteries (A) and veins (V) demonstrated moderate immunoreactivity to ERA. Lymph within lymphatic vessels (L) showed strong immunoreactivity to ERA. (**I**) Serosal interstitium (red arrowheads), and mesothelium (blue arrowheads) showed moderate and strong ERA immunostaining. Note, bordered area in (**A**) the negative control.

At 0 day of pregnancy, immunohistochemical localization of ERA in the rabbit cervix showed moderate cytoplasmic and nuclear immunostaining in both ciliated and secretory cells distributed randomly within the cervical lining epithelium. However, Most of the cervical lining epithelium showed negative immunostaining to ERA (Fig. 1A,B). The intraepithelial lymphocytes showed mild, moderate to strong cytoplasmic immunostaining to ERA (Fig. 1A,C,D). In addition, the subepithelial telocytes had moderate cytoplasmic and negative nuclear ERA immunoreactivity (Fig. 1E). Also, different kinds of cells within the lamina propria including fibroblasts and lymphocytes exhibited mild cytoplasmic and negative nuclear ERA immunoreactivity (Fig. 1F).

Mild immunoreactivity of ERA was expressed in the cytoplasm and nucleus of some of the smooth muscle fibers of the cervix (Fig. 1G). Moreover, the blood plasma, red blood cells and cytoplasm of leukocyte within the arteries and veins demonstrated moderate immunoreactivity to ERA. Lymph within lymphatic vessels also showed strong immunoreactivity to ERA. Also, some of the endothelial cells showed mild ERA immunostaining and the stroma surrounding the blood vessels exhibited mild and strong ERA immunostaining (Fig. 1H). Serosal interstitum and mesothelium showed moderate and strong ERA immunostaining (Fig. 1I).

### At 7 days of pregnancy

**Figure 2:**
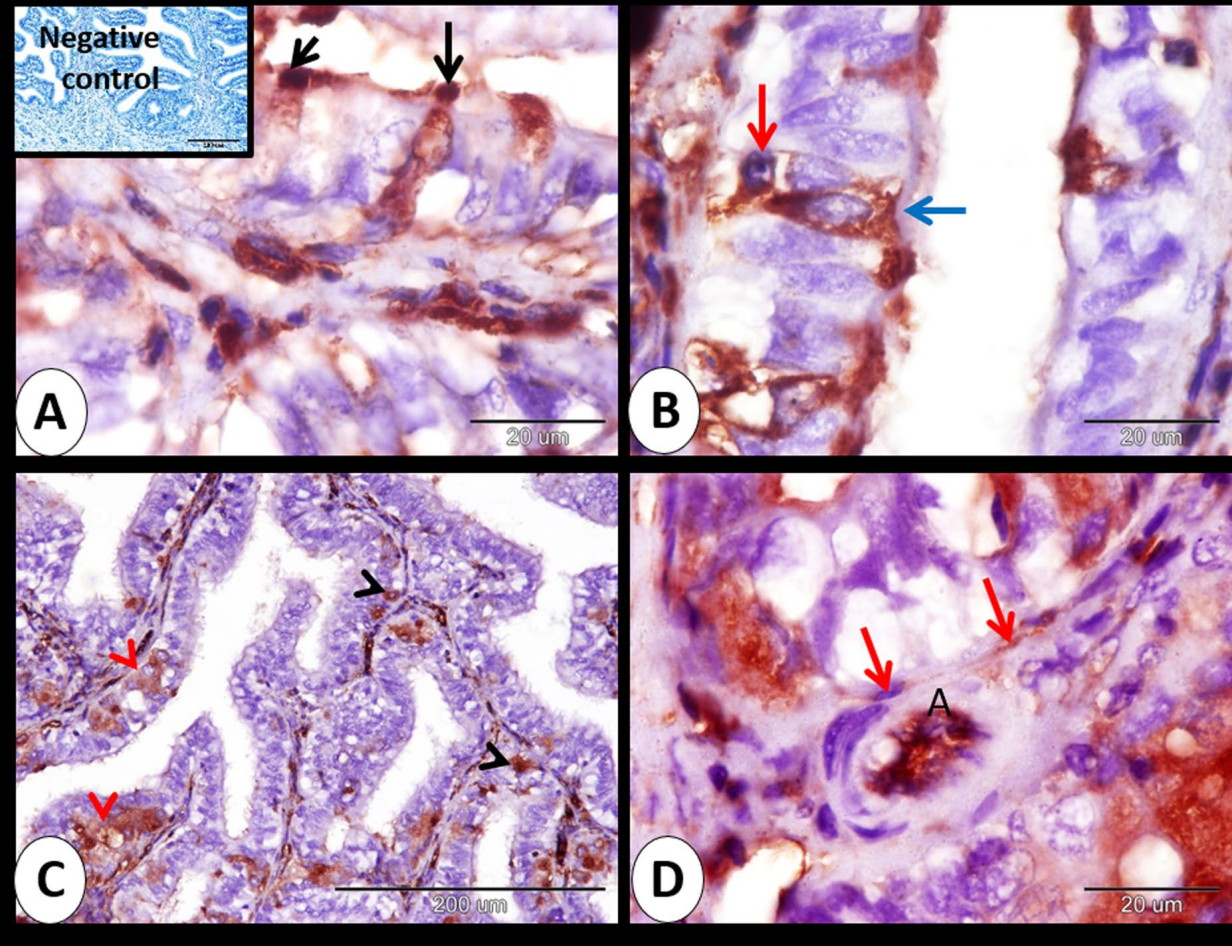
Immunohistochemical localization of ERA in rabbit cervix at 7 days of pregnancy. (**A**) secretory cells (black arrows) exhibited moderate cytoplasmic and moderate nuclear immunostaining to ERA. (**B**) ciliated cell (blue arrow) showed moderate cytoplasmic and negative nuclear immunostaining and intraepithelial lymphocytes (red arrow) showed moderate cytoplasmic ERA immunostaining. (**C**) intraepithelial neuroendocrine cells distributed singly (black arrowheads) or in groups (red arrowheads) expressed mild to strong cytoplasmic and nuclear immunostaining to ERA. (**D**) subepithelial telocytes (red arrows) showed moderate cytoplasmic and mild nuclear immunostaining to ERA and the artery endothelium (**A**) showed strong immunoreactivity to ERA. Note, bordered area in A the negative control.

At 7 days of pregnancy, moderate cytoplasmic and moderate nuclear immunostaining to ERA in secretory cells. However, ciliated cells exhibited moderate cytoplasmic and negative nuclear immunostaining. On the other hand, Most of the cervical lining epithelium showed negative immunostaining to ERA and positive cells distributed randomly within the cervical lining epithelium (Fig. 2A,B). The intraepithelial lymphocytes showed moderate cytoplasmic ERA immunostaining. Intraepithelial neuroendocrine cells expressed mild to strong cytoplasmic and nuclear immunostaining to ERA and distributed singly or in groups (Fig. 2C). In addition, the subepithelial telocytes showed moderate cytoplasmic and mild nuclear immunostaining to ERA (Fig. 2D).

**Figure 3:**
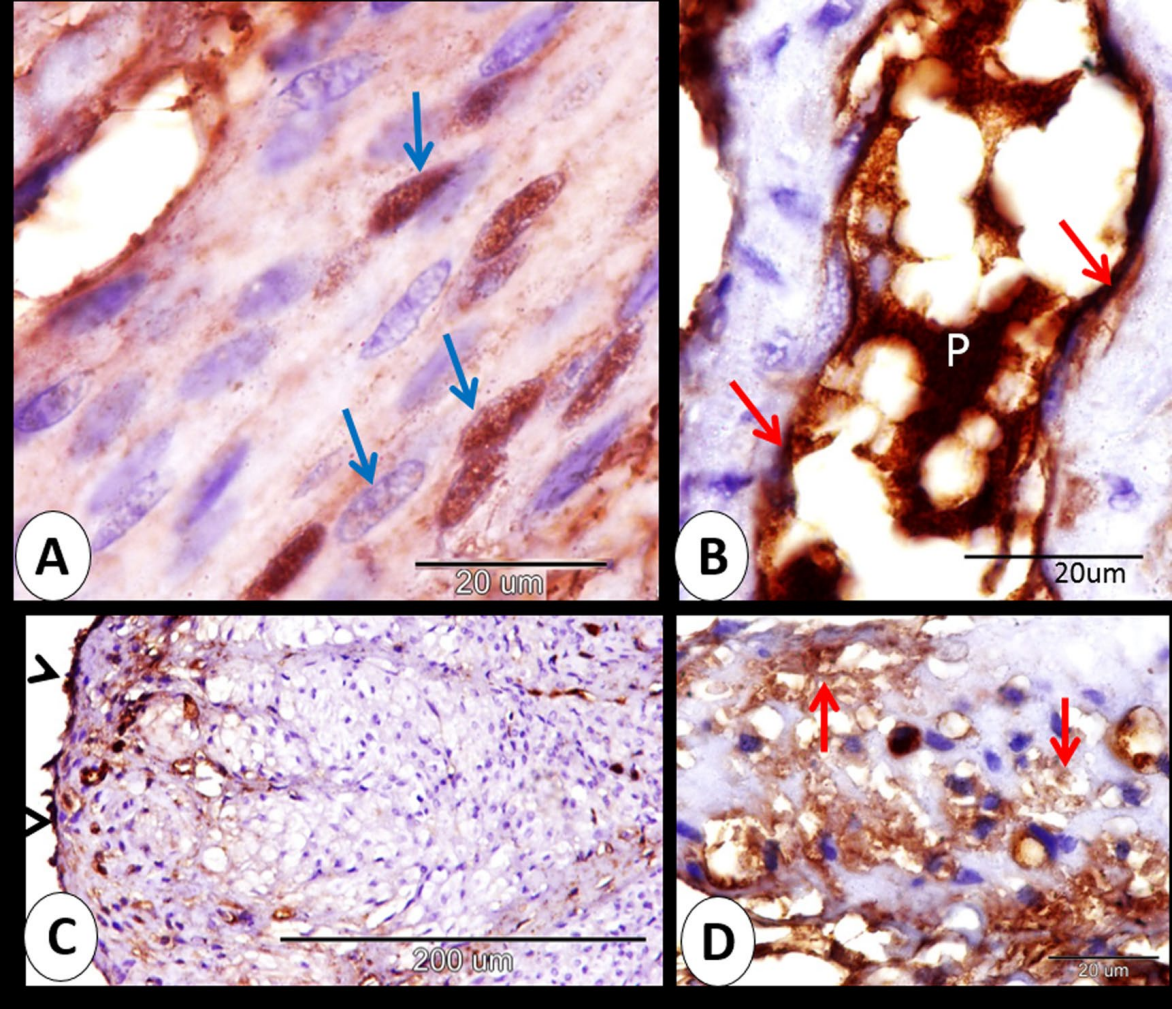
Immunohistochemical localization of ERA in rabbit cervix at 7 days of pregnancy. (**A**) smooth muscle fibers (blue arrows) showed mild cytoplasmic and moderate to strong nuclear immunoreactivity of ERA. (**B**) the endothelium of the blood vessels (red arrows) showed strong immunoreactivity to ERA and the blood plasma (P) demonstrated strong immunoreactivity to ERA. (**C**,**D**) Serosal mesothelium (black arrowheads) and serosal interstitium (red arrows) showed strong ERA immunostaining.

Our results indicated that mild cytoplasmic and moderate to strong nuclear immunoreactivity of ERA was expressed in some of the smooth muscle fibers of the cervix (Fig. 3A). Moreover, the endothelium of the blood vessels showed strong immunoreactivity to ERA and the blood plasma within the arteries and veins demonstrated strong immunoreactivity to ERA (Figs. 2D and 3B). Serosal interstitium and mesothelium showed strong ERA immunostaining (Fig. 3C,D).

### At 14 days of pregnancy

**Figure 4:**
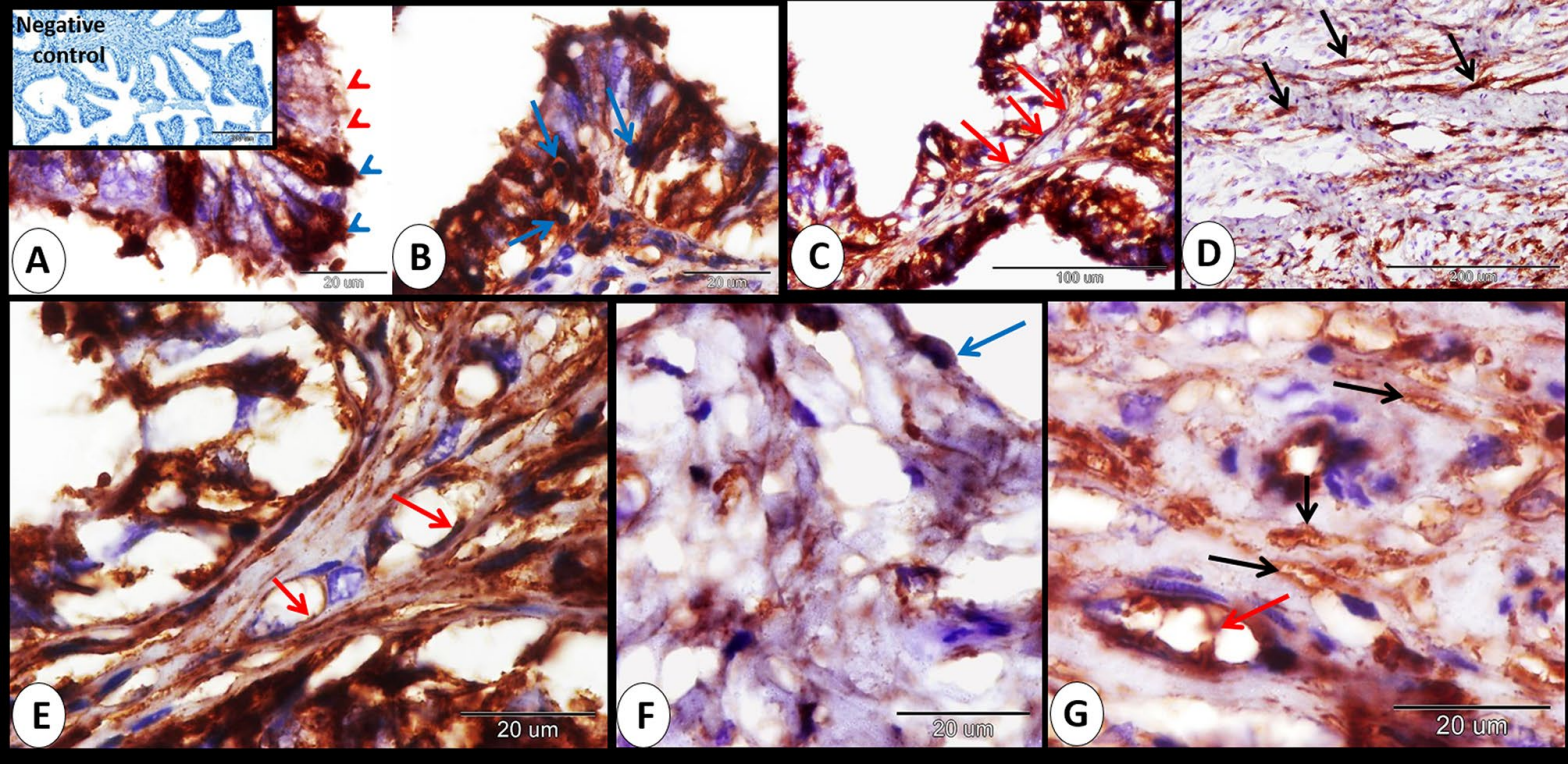
Immunohistochemical localization of ERA in rabbit cervix at 14 days of pregnancy. (**A**) secretory cells (red arrowheads) showed strong cytoplasmic and nuclear immunostaining to ERA and ciliated cells (blue arrowheads) exhibited mild cytoplasmic and nuclear immunostaining to ERA. (**B**) The intraepithelial lymphocytes (blue arrows) showed moderate to strong cytoplasmic and mild to moderate nuclear immunostaining to ERA. (**C**) Telocytes (red arrows) showed strong cytoplasmic and moderate nuclear reactions to ERA. (**D**) smooth muscle fibers (black arrows) demonstrated strong cytoplasmic and nuclear immunoreactivity of ERA. (**E**) the endothelial cells (red arrows) of the blood vessels exhibited moderate ERA immunostaining. (**F**) Serosal mesothelium (blue arrow) showed strong ERA immunostaining. (**G**) blood plasma (red arrow)demonstrated moderate immunoreactivity to ERA and interstitium (black arrows) showed strong immunoreactivity to ERA. Note, bordered area in A the negative control.

At 14 days of pregnancy, the rabbit cervix expressed strong immunostaining to ERA as a whole. Regarding the cervical lining epithelium, we observed that secretory cells demonstrated strong cytoplasmic and nuclear immunostaining to ERA but ciliated cells showed mild cytoplasmic and nuclear immunostaining to ERA (Fig. 4A). The intraepithelial lymphocytes indicated moderate to strong cytoplasmic and mild to moderate nuclear immunostaining to ERA (Fig. 4B). Telocytes in lamina propria showed a strong cytoplasmic and moderate nuclear reaction to ERA (Fig. 4C). Strong cytoplasmic and strong nuclear immunoreactivity of ERA was expressed in many of smooth muscle fibers of the cervix (Fig. 4D). Also, the endothelial cells of the blood vessels showed moderate ERA immunostaining (Fig. 4E). Serosal mesothelium showed strong ERA immunostaining (Fig. 4F). Also, blood plasma and interstitium showed moderate and strong immunoreactivity to ERA respectively (Fig. 4G).

### At 21 days of pregnancy

**Figure 5:**
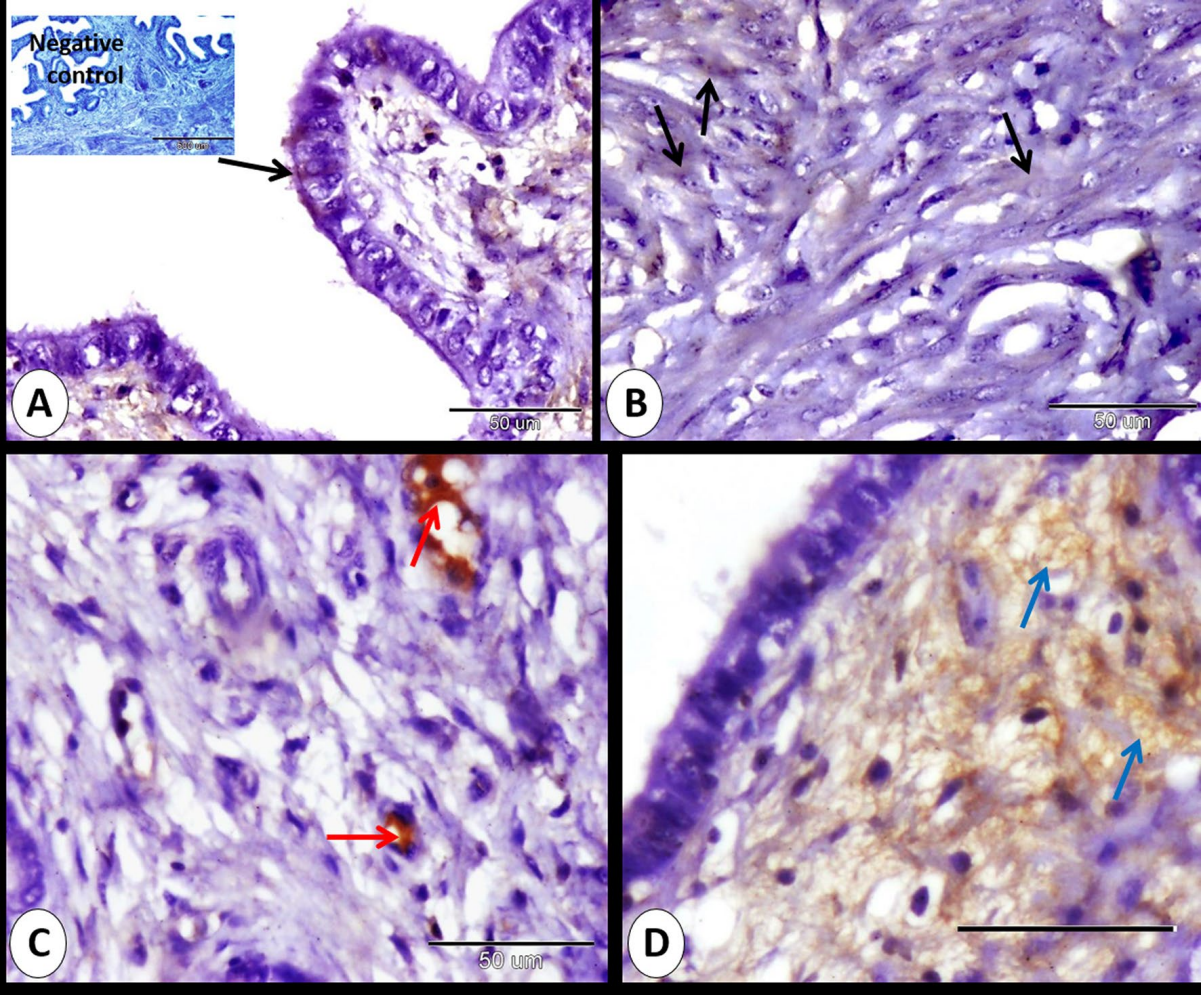
Immunohistochemical localization of ERA in rabbit cervix at 21 days of pregnancy. (**A**) Ciliated cells (black arrow) showed mild immunostaining to ERA. (**B**) Smooth muscle fibers (black arrows) exhibited mild immunostaining to ERA. (**C**) The blood plasma (red arrows) showed mild immunostaining to ERA. (**D**) Interstitium (blue arrows) showed mild immunostaining to ERA. Note, bordered area in **A** the negative control.

At 21 days of pregnancy, few ciliated cells and few smooth muscle fibers showed mild immunostaining to ERA. In addition, the blood plasma showed mild immunostaining to ERA. Interstitium exhibited mild immunostaining to ERA (Fig. 5A–D).

**Figure 6:**
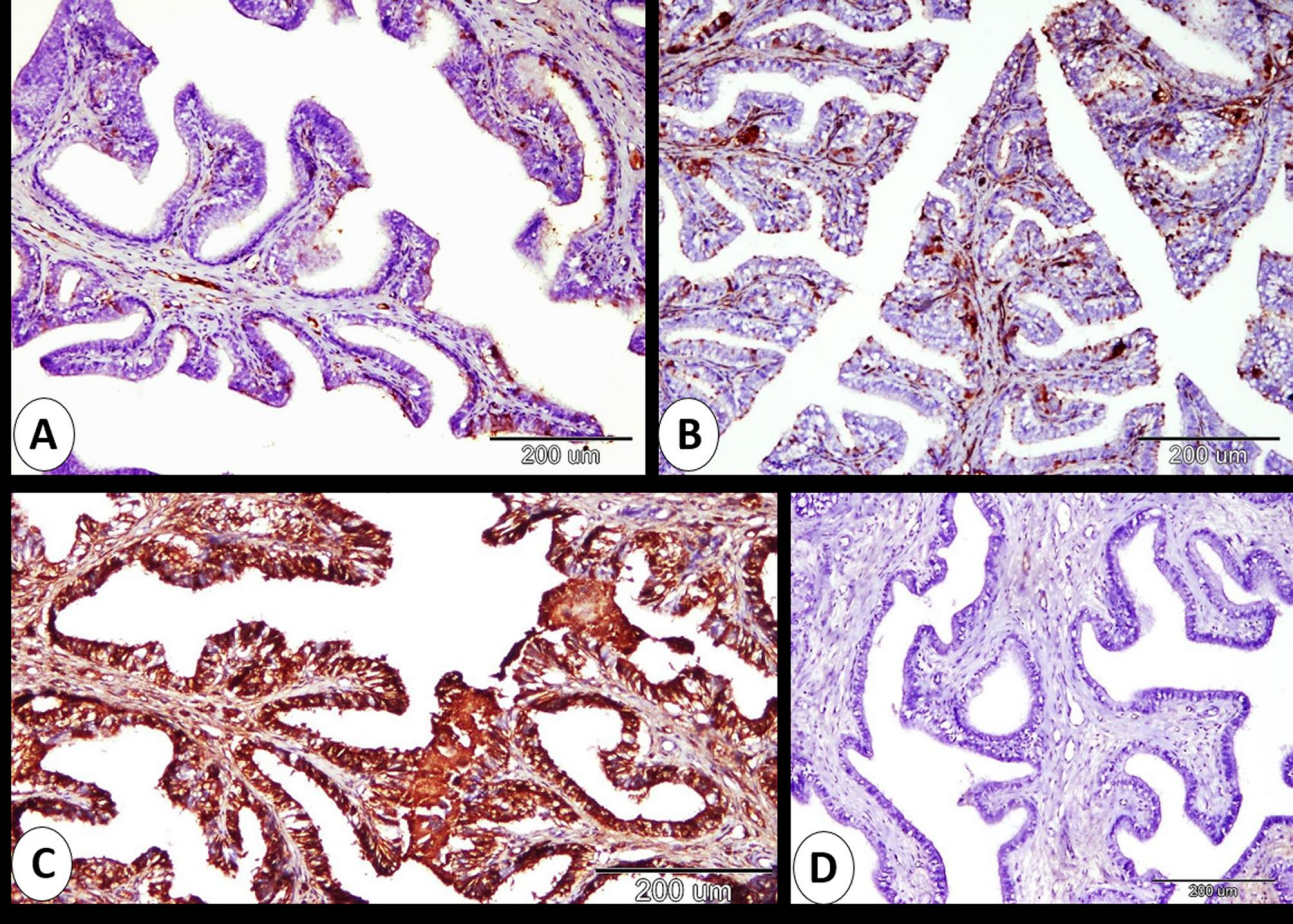
Immunohistochemical localization of ERA in rabbit cervix at 0, 7, 14 and 21 days of pregnancy. (**A**) Mild immunostaining of the rabbit cervix at 0 day of pregnancy. (**B**) Moderate immunostaining of the rabbit cervix at 7 days of pregnancy. (**C**) strong immunostaining of the rabbit cervix at 14 days of pregnancy (l). (**D**) mild immunostaining of the rabbit cervix at 21 days of pregnancy.

**Figure 7:**
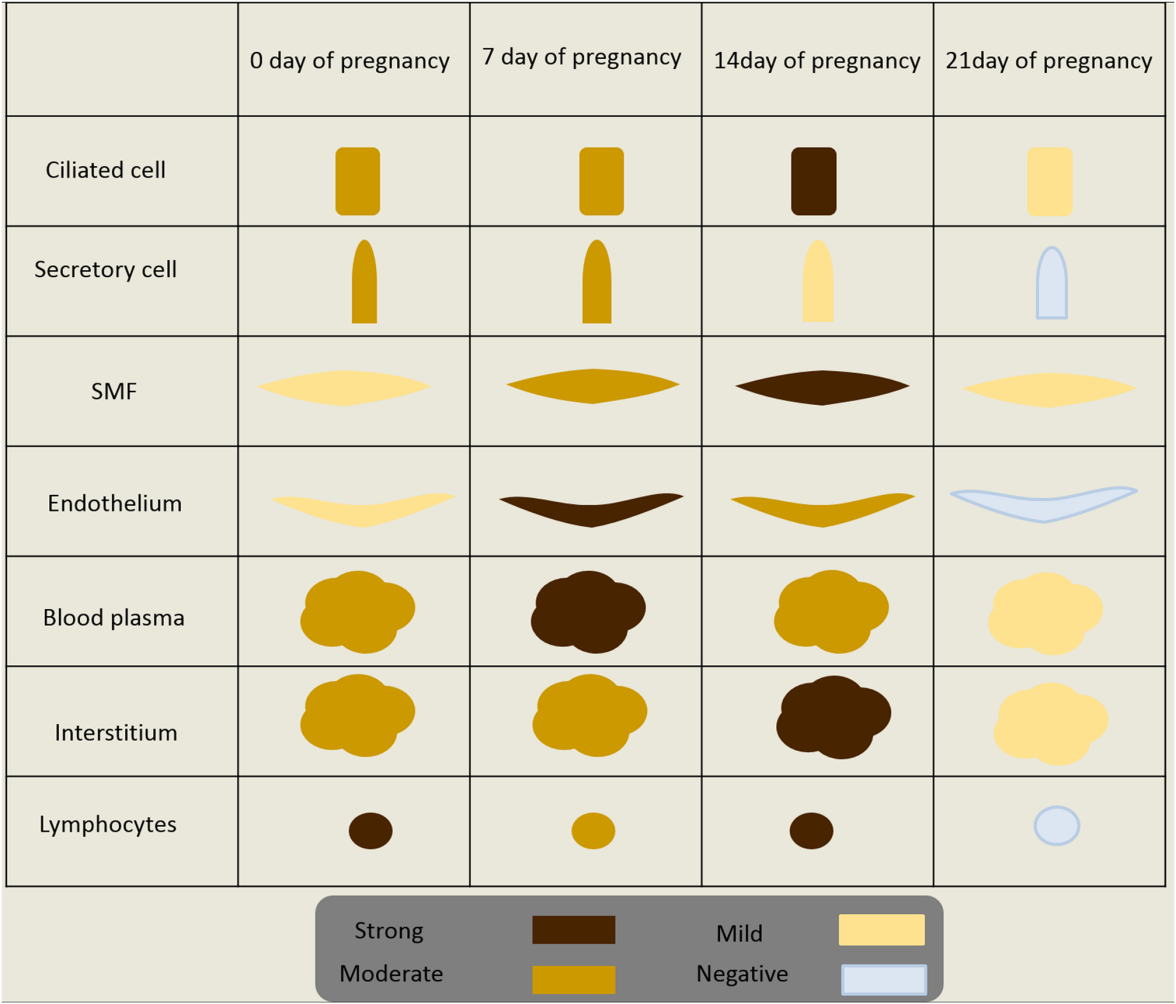
The intensity of the ERA immunoreaction at the different structural components of the cervix at different stages of pregnancy.

Overall, assessment of the immunoreactions indicated that the cervix of pregnant rabbits had different immunostaining to ERA at 0, 7, 14 and 21 days of pregnancy. The staining intensity was mild at 0 day, moderate at 7 days, strong at 14 days and mild at 21 days of pregnancy (Fig. 6A–D). Also, the summary of ERA immunoreaction occurred on different cervical structural components summarized in Fig. 7.

Percentage of ERA positive cells at different stages of pregnancy were demonstrated on Table 1.

**Table 1:** Percentage of immunohistochemical positive cells for ERA (Means ± SEM)**.**

Day of pregnancy/% of cell	0 day of pregnancy	7 day of pregnancy	14 day of pregnancy	21 day of pregnancy
Ciliated cells %	27.69 ± 0.55	33.33 ± 0.67	34.78 ± 0.28	22.91 ± 0.41
Secretory cells %	53.85 ± 0.53	83.33 ± 0.64	75.47 ± 0.14	–
Lymphocytes %	60.87 ± 0.20	65.00 ± 0.26	73.33 ± 1.86	–
Neuroendocrine cells %	–	65.43 ± 0.31	76.92 ± 0.90	–
SMFs %	35.29 ± 1.44	33.33 ± 0.33	68.52 ± 0.36	64.52 ± 0.29

## Discussion

Throughout pregnancy, the cervix has a load-bearing function to withstand the pressure from the growing fetus and not dilate during pregnancy^34^. Cervical ripening is a complex process occurring gradually to facilitate the expulsion of the fetus^35^. Several changes observed during these processes including cellular and extracellular matrix elements^36^. Moreover, cervical remodeling occurred through the modulating effects of estrogen receptors^37^. Also, at late pregnancy stages the cervical levels of the estrogen receptor are low^21^.

The present study demonstrated the presence of ERA in the rabbit cervix during pregnancy. Estrogen action is mediated primarily by binding to specific intracellular receptors in target cells^38^. Our study provides new information regarding the expression and localization of ERA in the normal rabbit cervix during different pregnancy stages. We determined that in the rabbit cervix, ERA displayed differences among cell types at 0, 7, 14 and 21 days of pregnancy. Based on the density of ERA, these may provide a favorable environment throughout pregnancy. Moreover, in the pregnant sheep and in women estrogen administration promotes the softening of the cervix under the experimental conditions^39^. However, the cervical softening in a pig not promoted by estrogen-only^40^.

The cervical lining epithelium of the rabbit consisted mainly of ciliated and secretory cells^22^. Our data revealed that ciliated cells exhibiting mild to strong immune staining to ERA and secretory cells showed negative to moderate immunostaining to ERA during different pregnancy stages. Estrogen was very important for the function of the ciliated cell during transport through the uterine tube and induce important secretory products of secretory cells^41^. The endocrine role of cervical lining epithelium preserves cervical function during pregnancy^42^.

Neuroendocrine cells observed on different parts of the female reproductive tract in non-pregnant conditions^29,43^. Our result indicated that neuroendocrine cells take an intraepithelial position and showed different immunoreactivity to ER.

Our data revealed than intraepithelial and subepithelial lymphocyte of the cervix during pregnancy showed from negative to strong positive immunoreactivity to ER. Estrogen plays a key role in the regulation of immune cells of the female reproductive tract^44^. Intraepithelial lymphocyte expresses positive immunoreactivity to ER in the uterine tube. Also, described previously in the management of local immune tolerance during the movement of sperm on the uterine tube^45^.

Our result indicated that stroma cells as fibroblasts showed immunoreactivity to ERA. Also, fibroblasts act as hormone receptors and through paracrine activity effect on epithelial cells^46^. Moreover, steroid hormones play an important role in the proliferation and differentiation of stromal cells and the maintenance of pregnancy. There were paracrine dialog between the epithelial lining of uterus and stroma cells controlled by steroid hormone receptors in these cells to regulate its function during pregnancy^47^.

Telocyte was newly discovered interstitial cell with paracrine activity and play a role in tissue repair and regeneration^33,48,49^. Also, it exhibited immunoreactivity to different steroid hormones^31,50,51^. Telocyte previously observed on the female reproductive tract^32,52–54^. Our results revealed that telocyte express moderate to strong immunoreactivity to ER during different stages of pregnancy. In addition, telocyte act as a steroid sensor on the female reproductive tract^55,56^.

The action of estrogen on smooth muscle was important for a successful pregnancy. Our data revealed that ERA expression on the smooth muscle fibers of the cervix increase gradually to reach the peak at mid-pregnancy then decreases at late pregnancy. These may be strongly related to the function of the cervix to be strongly closed during pregnancy and began to dilate at end of pregnancy which opposite which happened on the myometrium. The low expression estrogen receptor at early pregnancy help in myometrial quiescence and the increase of estrogen receptor expression until term pregnancy induce myometrial contraction during parturition these on the help of other hormones action^57^.

We described the expression of ERA at blood and lymph vessels during pregnancy which ranges between weak to strong. In addition, the role of estrogen described before on the blood vessels during pregnancy including growth and dilation to improve blood flow^58^. Also, in our results red and white blood cells showed immunoreactivity to ERA. Moreover, function of white blood cells was controlled by sex steroid hormones^59^. The expression of estrogen receptor on leukocytes endothelial cells of the cervical blood vessels were important in the regulation of white blood cells function in the induction of certain enzymes responsible for cervical remolding during pregnancy^60^. Also, the expression of ERA in RBCs observed by their effect on the function of RBCs as scavengers to reactive oxygen and nitrogen species^61^.

In the last few years, several studies began to focus on interstitium as fluid-filled spaces described on the gastrointestinal tract, dermis, and urinary bladder. Also, interstitium may be important on the mechanical structure of these organs and play a role in fibrosis and cancer metastasis^62^. In our result, the interstitium contains interstitial fluid which showed moderate immunostaining at early pregnancy, strong immunostaining at mid-pregnancy and mild immunostaining at late pregnancy for ERA these indicate the strong correlation between the interstitium and the cervical remodeling and cervical ripening. In addition, our data revealed that various fluid present on the cervix exhibiting immunoreactivity to ERA with different intensity throughout pregnancy including, blood plasma and lymph.

## Data Availability

The datasets used and/or analyzed during the current study are available from the corresponding author on reasonable request.

## References

[1] Wagner GP (2017). Evolution of gene expression in the uterine cervix related to steroid signaling: Conserved features in the regulation of cervical ripening. Sci. Rep..

[2] Zhao Y, Williams LM, Hannah LT, Ross AW, McKelvey WAC, Robinson JJ (1999). Oestrogen and progesterone receptor immunoreactivity and c-fos expression in the ovine cervix. Reproduction.

[3] Rodriguez HA (2003). Collagen remodelling in the guinea-pig uterine cervix at term is associated with a decrease in progesterone receptor expression. Mol. Hum. Reprod..

[4] Adashi EY, Rock JA, Rosenwaks Z (1996). Reproductive endocrinology, surgery, and technology.

[5] Pillai SB, Rockwell LC, Sherwood OD, Koos RD (1999). Relaxin stimulates uterine edema via activation of estrogen receptors: Blockade of its effects using ICI 182,789, a specific estrogen receptor antagonist. Endocrinology.

[6] Yan W, Chen J, Wiley AA, Crean-Harris BD, Bartol FF, Bagnell CA (2008). Relaxin (RLX) and estrogen affect estrogen receptor a, vascular endothelial growth factor, and RLX receptor expression in the neonatal porcine uterus and cervix. Reproduction.

[7] Winuthayanon W, Hewitt SC, Orvis GD, Behringer RR, Korach KS (2010). Uterine epithelial estrogen receptor α is dispensable for proliferation but essential for complete biological and biochemical responses. Proc. Natl. Acad. Sci..

[8] Re G (1995). Distribution of cytosolic oestrogen and progesterone receptors in the genital tract of the mare. Res. Vet. Sci..

[9] Wang H, Eriksson H, Sahlin L (2000). Estrogen receptors α and β in the female reproductive tract of the rat during the estrous cycle. Biol. Reprod..

[10] de Brito CP, de Oliveira CM, Soares FA, Faustino M, de Oliveira CA (2006). Immunohistochemical determination of estrogen receptor-α in vaginal and tumor tissues of healthy and TVT-affected bitches and their relation to serum concentrations of estradiol-17β and progesterone. Theriogenology.

[11] Xie Z, Shi H, Zhou C, Dong M, Hong L, Jin H (2007). Alterations of estrogen receptor-α and-β in the anterior vaginal wall of women with urinary incontinence. Eur. J. Obstet. Gynecol. Reprod. Biol..

[12] Rodríguez-Piñón M, Tasende C, Puime P, Garófalo EG (2008). Oestrogen and progesterone receptor binding capacity and oestrogen receptor alpha expression (ERα mRNA) along the cervix of cycling ewes. Reprod. Fertil. Dev..

[13] Hunter RE, Longcope C, Keough P (1987). Steroid hormone receptors in carcinoma of the cervix. Cancer.

[14] Lubahn DB, Moyer JS, Golding TS, Couse JF, Korach KS, Smithies O (1993). Alteration of reproductive function but not prenatal sexual development after insertional disruption of the mouse estrogen receptor gene. Proc. Natl. Acad. Sci..

[15] Meikle A, Garófalo EG, Rodríguez-Piñón M, Tasende C, Sahlin L (2001). Regulation by gonadal steroids of estrogen and progesterone receptors along the reproductive tract in female lambsActa. Vet. Scand..

[16] Vermeirsch H, Simoens P, Lauwers H (2000). Immunohistochemical detection of the estrogen receptor-α and progesterone receptor in the canine pregnant uterus and placental labyrinth. Anat. Rec..

[17] Tremblay GB (1997). Cloning, chromosomal localization, and functional analysis of the murine estrogen receptor β. Mol. Endocrinol..

[18] Bianchi CP, Sahlin L, Meikle A, Masironi B, Cavilla MV, Aba MA (2010). Endometrial population of oestrogen receptors alpha and beta and progesterone receptors A and B during the different phases of the follicular wave of llamas (*Lama glama*). Reprod. Domest. Anim..

[19] Couse JF, Korach KS (1999). Estrogen receptor null mice: What have we learned and where will they lead us?. Endocr. Rev..

[20] Breeveld-Dwarkasing VNA (2002). Immunohistochemical distribution of oestrogen and progesterone receptors and tissue concentrations of oestrogens in the cervix of non-pregnant cows. Reprod. Fertil. Dev..

[21] Stjernholm Y, Sahlin L, Malmström A, Barchan K, Eriksson HA, Ekman G (1997). Potential roles for gonadal steroids and insulin-like growth factor I during final cervical ripening. Obstet. Gynaecol..

[22] Mustafa F. E. Z. A. Histomorphological studies on the female reproductive tract inrabbit during pregnancy. Ph.D. Thesis. Fac. of Vet. Med.,Assuit Univ., Assuit, Eygpt (2018).

[23] Akerud A (2009). Uterine Remodeling During Pregnancy Studies on the Effect of Heparin/Heparan Sulfate.

[24] Mustafa FEZA, El-Desoky SM (2020). Architecture and cellular composition of the spleen in the Japanese Quail (*Coturnix japonica*). Microsc. Microanal..

[25] Mokhtar DM (2018). Cellular and stromal elements organization in the liver of grass carp, *Ctenopharyngodon idella* (Cypriniformes: Cyprinidae). Micron..

[26] Salama NM (2013). Immunohistochemical characterization of telocytes in ratuterus in different reproductive states. Egypt. J. Histol..

[27] Gosney JR, Sissons MC, Allibone RO (1988). Neuroendocrine cell populations in normal human lungs: A quantitative study. Thorax.

[28] Gunawardene AR, Corfe BM, Staton CA (2011). Classification and functions of enteroendocrine cells of the lower gastrointestinal tract. Int. J. Exp. Pathol..

[29] Czaja K, Sienkiewicz W, Vittoria A, Costagliola A, Cecio A (1996). Neuroendocrine cells in the female urogenital tract of the pig, and their immunohistochemical characterization. Cells Tissues Organs..

[30] Mustafa FEZA (2019). Putative primo-vascular system in rabbit placenta. J. Acupunct. Meridian Stud..

[31] Abdel-Maksoud FM, Abd-Elhafeez HH, Soliman SA (2019). Morphological changes of telocytes in camel efferent ductules in response to seasonal variations during the reproductive cycle. Sci. Rep..

[32] Abd-Elhafeez HH, Soliman SA (2017). New description of telocyte sheaths in the bovine uterine tube: An immunohistochemical and scanning microscopic study. Cells Tissues Organs.

[33] Abd-Elkareem M (2017). Cell-specific immuno-localization of progesterone receptor alpha in the rabbit ovary during pregnancy and after parturition. Anim. Reprod. Sci..

[34] Oxlund BS, Ørtoft G, Brüel A, Danielsen Cl C, Bor P, Oxlund H, Uldbjerg N (2010). Methodology Collagen concentration and biomechanical properties of samples from the lower uterine cervix in relation to age and parity in non-pregnant women. Reprod. Biol. Endocrinol..

[35] Calder AA. 1994. The cervix during pregnancy. In *The Uterus* (Chard, T. & Crudzinskas, J.G. eds) Ch.13: 288–207.

[36] Danforth DN (1947). The fibrous nature of the human cervix, and its relation to the isthmic segment in gravid and nongravid uteri. Am. J. Obstet. Gynecol..

[37] Wang H, Stjernholm Y, Ekman G, Eriksson H, Sahlin L (2001). Different regulation of oestrogen receptors α and β in the human cervix at term pregnancy. Mol. Hum. Reprod..

[38] Gronemeyer H (1992). Control of transcription activation by steroid hormone receptors. FASEB J..

[39] Gordon AJ, Calder AA (1977). Oestradiol applied locally to ripen the unfavourable cervix. Lancet.

[40] Winn RJ, Baker MD, Sherwood OD (1994). Individual and combined effects of relaxin, estrogen, and progesterone in ovariectomized gilts. I. Effects on the growth, softening, and histological properties of the cervix. Endocrinology.

[41] Li S (2017). Estrogen receptor α is required for oviductal transport of embryos. FASEB J..

[42] Andersson S, Minjarez D, Yost NP, Word RA (2008). Estrogen and progesterone metabolism in the cervix during pregnancy and parturition. J. Clin. Endocrinol. Metab..

[43] Wanke, I. E. Reproduction and the APUD system. In *Seminars in surgical oncology*, Vol. 9, No. 5, 394–398 (Wiley, New York, 1993). 10.1002/ssu.2980090508 7902608

[44] Lee SK, Kim CJ, Kim DJ, Kang JH (2015). Immune cells in the female reproductive tract. Immune Netw..

[45] Ulziibat S (2006). Identification of estrogen receptor β-positive intraepithelial lymphocytes and their possible roles in normal and tubal pregnancy oviducts. Hum. Reprod..

[46] Perrot-Applanat M, Deng M, Fernandez H, Lelaidier C, Meduri G, Bouchard P (1994). Immunohistochemical localization of estradiol and progesterone receptors in human uterus throughout pregnancy: Expression in endometrial blood vessels. J. Clin. Endocrinol. Metab..

[47] Pawar S, Laws MJ, Bagchi IC, Bagchi MK (2015). Uterine epithelial estrogen receptor-α controls decidualization via a paracrine mechanism. Mol. Endocrinol..

[48] Popescu LM, Hinescu ME, Ionescu N, Ciontea SM, Cretoiu D, Ardeleanu C (2005). Interstitial cells of Cajal in pancreas. J. Cell Mol. Med..

[49] Bei Y, Wang F, Yang C, Xiao J (2015). Telocytes in regenerative medicine. J. Cell Mol. Med..

[50] Hutchings G, Williams O, Cretoiu D, Ciontea SM (2009). Myometrial interstitial cells and the coordination of myometrial contractility. J. Cell Mol. Med.

[51] Cretoiu SM, Cretoiu D, Simionescu AA, Popescu LM (2012). Telocytes in human fallopian tube and uterus express estrogen and progesterone receptors. Sex Steroids..

[52] Popescu LM (2005). Novel type of interstitial cell (Cajal-like) in human fallopian tube. J. Cell Mol. Med..

[53] Hutchings G, Deprest J, Nilius B, Roskams T, De Ridder D (2006). The effect of imatinib mesylate on the contractility of isolated rabbit myometrial strips. Gynecol. Obstet. Investig..

[54] Cretoiu SM, Cretoiu D, Marin A, Radu BM, Popescu LM (2013). Telocytes: Ultrastructural, immunohistochemical and electrophysiological characteristics in human myometrium. Reproduction.

[55] Cretoiu SM, Cretoiu D, Suciu L, Popescu LM (2009). Interstitial Cajal-like cells of human Fallopian tube express estrogen and progesterone receptors. J. Mol. Histol..

[56] Klein M, Urban L, Deckov I, Danisovic L, Polak S, Danihel L, Varga I (2017). Distribution of telocytes in the corpus and cervix of human uterus: An immunohistochemical study. Biologia.

[57] Condon JC, Kyathanahalli C, Anamthathmakula P, Jeyasuria P (2019). Estrogen/estrogen receptor action and the pregnant myometrium. Curr. Opin. Physiol..

[58] Leiberman JR, Wiznitzer A, Glezerman M, Feldman B, Levy J, Sharoni Y (1993). Estrogen and progesterone receptors in the uterine artery of rats during and after pregnancy. Eur. J. Obstet. Gynecol. Reprod. Biol..

[59] Stygar D, Masironi B, Eriksson H, Sahlin L (2007). Studies on estrogen receptor (ER) a and b responses on gene regulation in peripheral blood leukocytes in vivo using selective ER agonists. J. Endocrinol..

[60] Stygar D, Wang H, Vladic YS, Ekman G, Eriksson H, Sahlin L (2001). Co-localization of oestrogen receptor β and leukocyte markers in the human cervix. MHR Basic Sci. Reprod. Med..

[61] Vona R (2019). Functional estrogen receptors of red blood cells. Do they influence intracellular signaling?. Cell Physiol. Biochem..

[62] Benias PC (2018). Structure and distribution of an unrecognized interstitium in human tissues. Sci. Rep..

